# Participatory Design of an Electronic Cross-Facility Health Record (ECHR) System for Pediatric Palliative Care: A Think-Aloud Study

**DOI:** 10.3390/children8100839

**Published:** 2021-09-24

**Authors:** Theresa Sophie Busse, Chantal Jux, Sven Kernebeck, Larissa Alice Dreier, Dorothee Meyer, Daniel Zenz, Boris Zernikow, Jan Peter Ehlers

**Affiliations:** 1 Department of Didactics and Educational Research in Health Science, Faculty of Health, School of Medicine, Witten/Herdecke University, 58448 Witten, Germany; chantal.jux@uni-wh.de (C.J.); sven.kernebeck@uni-wh.de (S.K.); jan.ehlers@uni-wh.de (J.P.E.); 2 PedScience Research Institute, 45711 Datteln, Germany; l.dreier@pedscience.de (L.A.D.); d.meyer@pedscience.de (D.M.); b.zernikow@kinderklinik-datteln.de (B.Z.); 3 Department of Children’s Pain Therapy and Pediatric Palliative Care, Faculty of Health, Witten/Herdecke University, 58448 Witten, Germany; 4 Smart-Q Softwaresystems GmbH, 44801 Bochum, Germany; zenz@smart-q.de; 5 Pediatric Palliative Care Centre, Children’s and Adolescents’ Hospital, 45711 Datteln, Germany

**Keywords:** palliative care, pediatrics, electronic health record, think-aloud, electronic medical record, software development, codesign, participatory design, shared health record

## Abstract

Background: Pediatric palliative care (PPC) patients experience years of multisectoral and professional care. An electronic cross-facility health record (ECHR) system can support the immediate exchange of information among PPC professionals. Based on a needs assessment, a prototype ECHR system was developed. Methods: To evaluate potential users’ perspective regarding the system, a qualitative observational study was conducted consisting of a concurrent think-aloud session and a semi-structured qualitative interview. Results: Twenty PPC professionals (nurses, physicians) from specialized outpatient PPC teams, a PPC unit, and medical offices rated the ECHR system as a helpful tool to improve the exchange and collection of information, communication between PPC professionals, and treatment planning. From the user’s point of view, the basic logic of the ECHR system should be further adapted to improve the interaction of data remirrored from patient records of outpatient and inpatient care with those entered via the system. The users wished for further functions (text search) and content (information on therapies). Some content, such as the treatment process, needs to be further adapted. Conclusion: The developed ECHR system needs to be more specific in some features by offering all available information; while for other features, be less specific to offer a quick overview. The ability to share information promptly and automatically was seen as a tremendous improvement to the quality of care for PPC patients.

## 1. Introduction

Pediatric palliative care (PPC) represents a holistic care approach for children, adolescents, and young adults with life-limiting or life-shortening illnesses [1]. These mainly include neurologic, genetic/congenital, and neuromuscular, but also metabolic conditions. Some illnesses are rare or even unknown and accompanied by a variety of complex symptoms, such as agitation, seizures, or sleep disorders. The children are often cognitively impaired or unable to express themselves verbally due to the diseases [2,3,4,5].

A crucial difference between PPC and adult palliative care patients is the necessity of PPC for many years for children [6]. Due to their variable general conditions and to relieve the family’s burden, PPC patients receive (concurrent) care from a variety of inpatient and outpatient providers [7]. Depending on the current symptomatology and the needs of the family and patients, different PPC professionals may be involved in the care, with a physical, psychological, social, or spiritual focus depending on the profession [8]. In stable phases of their conditions, PPC patients in Germany are cared for at home. The families are supported by pediatricians and general practitioners from medical offices, as well as general outpatient PPC teams. The support of specialist physicians and various therapists (e.g., occupational therapy, physiotherapy, speech therapy) and specialized outpatient PPC (SOPPC) teams can be of further assistance to the patients and their families. There is also the possibility of additional support from outpatient hospice services, where trained volunteers accompany the families. Additionally, admission to a pediatric hospice is an option. Pediatric hospices are facilities in which patients and their families can be accompanied by a multiprofessional team during the entire course of the disease and beyond death. The stays usually last four weeks, during which the PPC professionals at the hospice take over the care of the patients, thus relieving the family. It is also possible to carry out a final accompaniment in the children’s hospice. In the event of symptom exacerbation, planned or acute admission to a PPC unit (PPCU) may occur [6]. The arrangements for PPC care vary regionally and nationally, according to finances, number of trained professionals, and the awareness for the necessity of PPC [9].

Information about treatments, current situations, and future treatment approaches is therefore documented and needs to be shared among all PPC professionals [10]. For example, healthcare professionals from the PPC of oncology patients reported in one study that the tasks of PC teams and oncology teams would often overlap [11]. This is where looking at documentation could help to see what activities have already been done. Nurses in the study also indicated that they felt symptoms were often not mentioned to the oncologist [11]. This indicates how important it is that, for example, symptom documentation is shared with other PPC professionals. Evidence suggests that PPC professionals desire a way to share common information and communicate with each other [10,12]. Shared electronic health records allow easy access to patient information, a reduced risk of treatment errors, an increased efficiency of professionals, and a reduction of national healthcare costs [13]. The shared electronic health records that currently exist in Germany only serve to transfer files and are case related (e.g., for a hospital to transfer information from the hospital to the general practitioner) [14,15,16]. This existing concept is not sufficient for PPC: professionals want to work collaboratively on documents and have the possibility to view the documentation history of all involved PPC professionals since diagnosis of a life-limiting illness. The use of a shared electronic cross-facility health record (ECHR) with the ability to share content throughout the whole treatment of a life-limiting illness may be beneficial in this regard. To the best of our knowledge, such ECHRs do not exist in Germany. As a result, information sharing between the different care settings is unstructured and time consuming. Failure to share information can result in PPC professionals not having all the information, leading to treatment errors or unnecessary double documentation and interventions.

A common problem in the development and introduction of digital documentation systems is that they do not adequately address user needs [17]. Additionally, they do not support clinical workflows [18] or are characterized by poor usability [19]. To minimize these difficulties and increase acceptance, approaches in which systems are developed in a participatory design (originally co-operative design) process with future users are suitable [20,21]. Participatory design is a “design process where both users and designers are participating actively and creatively, drawing on their different qualifications” [22].

This study is part of the ELSA-PP (Electronic Intersectoral Record System for Palliative Care) Project (see Appendix A) in which a prototype ECHR was developed. The participatory design followed the design thinking model consisting of the following steps: (1) empathy, (2) define, (3) ideate, (4) prototype, (5) test, and (6) iterate. The exact procedure is described in a related paper [16]. This article describes that, in terms of iteration, the software previously tested as a prototype was programmed and then tested with potential future users. For this purpose, the proven methodology of concurrent think-aloud (CTA) was used. In CTA, participants are asked to verbalize their thoughts while using software [23,24]. Using CTA made it possible to capture the cognitive and emotional reactions and processes of participants while using software [25].

The aim of this study was to evaluate how potential users from the PPC setting perceive the prototype ECHR system and to identify their wishes related to an adaptation of the prototype.

## 2. Methods

### 2.1. Study Design

A qualitative observational study was conducted with a CTA method oriented on the approach of Boren and Ramey [26], followed by a semi-structured interview. Following a task-oriented approach [27], participants were asked to explore the ECHR system while performing tasks that corresponded to the requirements of their clinical everyday lives. The tasks (File S1) were designed to test the ECHR system components for functionality, content, and logic. To stimulate the imagination of participants, a fictional patient was used [28]. The ECHR system was filled with dummy data to design a scenario as realistically as possible [29].

An interview guide (File S2) was developed through discussion and consensus building among the research and development team and followed a pretest with a PPC professional. This aimed to assess overall feedback on the ECHR, independent of the specific tasks. The interview guide also included questions on whether any content or functionality was redundant or missing and what potential impact the ECHR system might have on the communication of PPC professionals.

### 2.2. Prototype ECHR

The prototype ECHR (hereafter ECHR) system in this study was developed in the ELSA-PP project, including a comprehensive needs assessment and conceptualization phase [16].

Basically, the ECHR system is a web-based system that can be accessed on the internet in real-time of PPC professionals who are given personal access to a patient’s ECH. Moreover, it is going to be linked to the electronic health records used at the PPCU and SOPPC and to be bidirectional synchronized. This should make it possible to display information collected on the PPCU and information collected in the context of the SOPPC together and to have it supplemented by other PPC professionals. For example, it is possible to see directly that a diagnosis was made in the hospital and the pediatricians can enter which medication they prescribed for it at home. This in turn can be viewed by all other PPC professionals.

Direct entries in the ECHR system can only be made in specific areas (personal notes, user-specific calendar, contact history, medication, patient-related calendar, treatment process) by users. In some areas, the option to upload documents is given (see below). The reason for this was that remirroring data from the ECHR system to the SOPPC and/or PPCU systems could result in data being lost. For example, if someone enters a drug into the ECHR that is rejected by the SOPPC team, it will not appear in the SOPPC drug schedule. Due to the mirroring between the SOPPC and ECHR systems in real time, the medication order is subsequently also removed from the ECHR system, and the order is no longer traceable. This logic is additionally responsive to user requirements in the previous requirements analysis, based on the assumption that SOPPC teams and PPCUs should be involved in medication decisions, thus providing assurance.

Prospectively, to use a patient’s ECHR, the various stakeholders involved in the patient’s care process must be authorized to use it.

**Figure 1:**
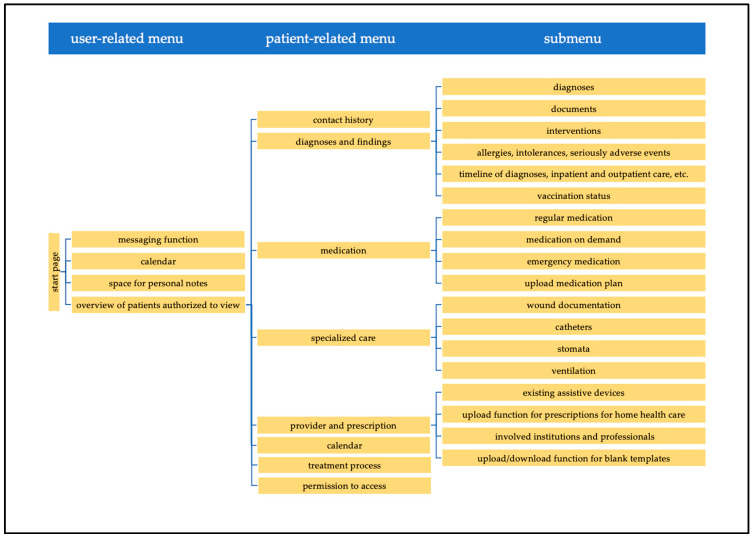
Structure of the ECHR system.

**Figure 2:**
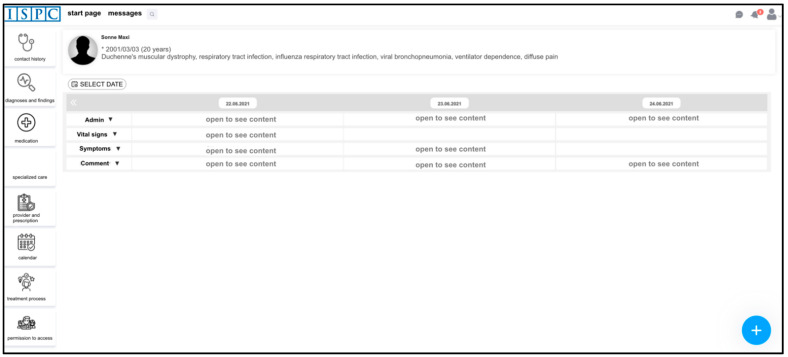
Screenshot from the patient-related menu and contact history (translated).

The ECHR system is structured as follows (Figure 1):

After logging in, the system’s **start page** offers an overview of and access to information of all patients for whom a corresponding PPC provider (“user” in the following) is authorized to view. In addition, it is possible to enter personal notes and save appointments in a calendar, which can only be viewed by the respective user. After a patient is selected, one will be brought to the patient’s ECHR. At the top of the view, one will find information about the patient. On the left, there are all the different contents to choose from, which are described in more detail below (Figure 2).

The **contact history** contains all documentations of contacts (home visits, phone calls, etc.) between users and the patients and their families or between users among each other in chronological order. Contacts that are documented in the PPCU or the SOPPC systems are transferred. With the help of an online form, it is possible to document with whom a contact has taken place and what was discussed. In addition, specific documentation of vital parameters, symptoms, physical examination, psychosocial information, and tasks to be completed can be viewed. In the view of all documented contacts, a field is displayed at the top, in which PPC professionals are able to see who made an entry and the date, time, and in which health record system (PPCU system, SOPPC system, or ECHR) it was made. To reduce loading times, all documented content is initially collapsed and not directly visible.

The **diagnoses and findings** view includes the following: (a) an overview of all of a patient’s diagnoses; (b) the possibility to upload and view different documents sorted by different tags (e.g., lab results); (c) an overview of a patient’s allergies, intolerances, and serious adverse events; (d) information about vaccination status; and (e) a view where all diagnoses, inpatient stays, outpatient enrollments, events, documented contacts, and appointments are arranged in a timeline.

The **medication** view is divided into regular medication, medication on demand, and emergency medication. In addition, it is possible to check the patient’s current medication plan, add orders, and discontinue medication. Users can upload medication plans from their own system. When a new medication plan has been uploaded in the ECHR system, a red banner appears, indicating an updated medication plan is available. In this view, it is possible to make suggestions for medication changes, which need to be confirmed by PPC professionals of the SOPPC team or the PPCU and were previously only visible as suggestions.

The **provider and prescription** view includes the following: (a) information on assistive devices a child uses; (b) which persons and institutions are involved in the PPC process; (c) ways to upload prescriptions for home health care; and (d) a section where blank templates can be uploaded.

In the **patient-related calendar**, a patient’s appointments can be entered.

The **treatment process** view enables symptom-related documentation of treatment planning. For each symptom, hypotheses, correspondingly initiated interventions, and their effects can be documented. The treatment process thus offers an overview of treatment and the traceability of treatment decisions for all involved PPC professionals.

Through the **messaging function**, messages can be sent to other PPC professionals caring for the same patient.

The **specialized care** section includes: (a) the option to view wound documentation; (b) a section where catheters can be documented with the respective duration and additional information (Charière); (c) information on tracheostoma, ileostoma, urostoma, and colostoma; and (d) information about ventilation machines and ventilation parameters as well as whether oxygen is administered.

In the **permission to access** section, it is possible to see which persons have access to the ECHR, which areas these persons can see, and where they can make entries.

### 2.3. Participants

As potential future users of the ECHR system, people from the PPCU, SOPPC teams, and medical offices were asked to take part in the study. In total, 27 PPC professionals from the PPCU, 23 PPC professionals from three SOPPC teams, three PPC professionals working in the PPCU as well as on a SOPPC team, and 177 general practitioners and pediatricians from medical offices were asked to participate. The numbers of recruited persons are based on the fact that within the framework of the ELSA-PP project, the employees of one PPCU and three SOPPC teams had agreed to participate and were therefore asked to take part in this project step. In addition, physicians who regularly work on the PPCU, and therefore regularly provide care for PPC patients, were contacted.

The nurses from the PPCU were recruited with the help of a poster placed on the message board of the PPCU. In addition, attention was drawn to the project at the beginning of some shift handovers. Recruitment of physicians from the PPCU and all PPC professionals from the SOPPC teams were asked by email to participate. Physicians from physician practices were contacted by phone, mail, or email. All professionals contacted had experience with PPC patients. For participation, a monetary compensation of 40€ per hour was disbursed.

All participants gave their informed consent prior to participation.

### 2.4. Data Collection and Procedure

Data collection took place in May and June 2021 (duration of seven weeks). The sessions were conducted by members of the research and development team (L.A.D., S.K., D.M., and T.S.B.) with good proficiency in qualitative research. Data collection was conducted until data saturation was reached.

During each session, two researchers were present, with one conducting the session and the other taking notes. Audio and screen movements were recorded via screencast using the software Captura (version 8.0). Two variations of the sessions were offered to allow participants the greatest possible flexibility and sense of security in the COVID-19 pandemic:

(1) Remote sessions

Participants shared their screens using a video conferencing tool (Zoom.us, version 5.4.7) and could click through the ECHR system themselves while the researchers watched.

(2) Face-to-face sessions

These sessions took place in a standardized regular office. The hardware setup included a desktop computer mirrored with a monitor so that the interviewers could observe the participants’ actions while keeping their distance. For audio recording, an external microphone was used (Auna MIC-900B).

Prior to the CTA sessions, the tasks were sent to the participants (remote session) or printed out as leaflets (face-to-face CTA sessions). All participants received the same guidance.

In the beginning of the session, participants were informed about the CTA procedure. It was emphasized that there was neither a right nor a wrong way to complete the tasks but that it was rather a question of testing the ECHR system from their perspective [29]. A brief introduction to the ECHR system and its basic logic was given (File S3). Participants were also informed that they should first try to solve problems on their own and verbalize this accordingly [23]. Some of the system views were not self-explanatory due to their unique logic. Therefore, at some points, the interviewers explained the view and its functionality, content, and/or logic supported by a previously developed guideline (File S1).

After the introduction, participants performed the tasks that they should first read out loud and then verbalized their actions and thoughts while performing them to gather their subjective impressions [30].

To avoid making the CTA sessions too long, not all of the ECHR system’s core components were tested (excluding specialized care and permission to access). In addition, the completion of the modules by the software company would have required more time. Waiting for this would have further delayed the entire development of the ECHR system.

In one remote session, a participant could not control the software himself due to unsolvable technical issues (18_physician_MO). The participant was guided through the software using the tasks, while the interviewers shared their screen and performed the clicking. Nevertheless, various content-related focal points were addressed in this CTA session, which is why it was included in the evaluation, and the special situation was considered.

### 2.5. Data Analysis

The CTA session audio recordings were transcribed verbatim (transcription rules of Dresing and Pehl; [31]) and analyzed by means of structuring qualitative content analysis using MAXQDA Standard 2020 software [32]. For this, first, a deductive approach was chosen to achieve sorting of the results according to the tasks. This was followed by an inductive approach to code the data material within the main categories (MCs). The time needed for executing a task was not scored because of the open and exploratory approach.

Two independent researchers (T.B., C.J.) performed the analysis. A category system was developed, reviewed, and discussed by the study’s other researchers (D.M., L.A.D., S.K.) until a consensus was reached.

The original quotes were translated into English. All participants were assigned pseudonyms following the structure “interview number_profession_setting” (e.g., 03_physician_PPCU).

## 3. Results

Twenty PPC professionals participated in the study (Table 1). All participants had different levels of experience with electronic documentation. Each session lasted between 43 and 114 min (average of 74.6 min). Ten sessions were remote sessions, and ten sessions were conducted as face-to-face sessions

**Figure 3:**
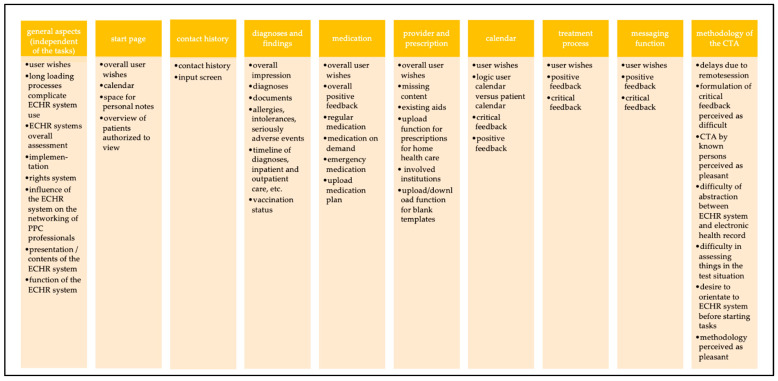
Overview of the main categories and subcategories.

A total of 1175 codes were assigned (range: 25 to 89 codes per interview). Ten MCs emerged: (1) “general aspects (independent of the tasks)”, (2) “start page”, (3) “contact history”, (4) “diagnoses and findings”, (5) “medication”, (6) “provider and prescription”, (7) “calendar”, (8) “treatment process”, (9) “messaging function”, and (10) “methodology of the CTA”. Subcategories were formed for each MC, which mostly represented the associated elements or functions (e.g., “medication” with subcode “emergency medication”) (Figure 3).

The representations of the results are structured into statements regarding functions, content, and logic as resolved in the analysis. Due to the extensive number of participants feedback, a selection of the participant feedback is described in the following sections. A table with the detailed suggestions and critic is provided in the appendix (File S4).

### 3.1. MC 1—General Aspects (Independent of the Tasks)

Concerning the functions of the ECHR system, the participants wished for the possibility of a free text search and the option to expand and collapse all fields simultaneously in several places. Some participants from medical offices also indicated that they would like to have the ability to automatically transfer data into and from their system. However, at the same time, they were aware of the challenges due to interoperability standards.

In accordance with the content of the ECHR system, the risk of information overload was named at various points. Participants feared this would result in content from other health records being transferred, unfiltered, and duplicated within the ECHR.

In terms of logic, there were some aspects that were criticized in several areas of the ECHR system. First, participants stated that in some areas in the ECHR system, they were not able to add content, and the only content that was displayed was fed from PPCU and SOPPC teams’ documentation. Although the intention here was to avoid clutter and the risk of overwriting data, users indicated that they wanted to have the option of adding information like diagnoses or allergies. Second, the permissions system was something that concerned the participants. They wished to be able to activate areas for individual patients and to inactivate other areas. Some of them also mentioned that not all persons should be allowed to make all types of entries and changes.

*“But what I had already said is that you really have to look at who has access to what and who can write something where. That not everyone has their fingers in the pie, but that there really are things, okay, now only the nursing management can make entries. And there may be only doctors, and the same is true with access to certain things. (08_nurse_PPCU)”*

One issue that was frequently named was that of jurisdiction. Participants were unsure who should ultimately make decisions and entries and had a desire for a PPC professional to be responsible for maintaining the ECHR system.

Regarding the functions of the ECHR system, many of the participants felt the loading times were too long and annoying; sometimes, they did not even recognize that something was loading. Therefore, they wished for a visual notice. Additionally, the participants wished for reminders when an appointment took place, or a document had to be updated. They wished that they could decide individually, depending on the patient, whether to be notified by email of new entries in the ECHR system. The majority of participants rated the ECHR system positively in terms of its ability to improve communication with other PPC professionals, to improve time savings, to provide a better overview of a patient’s overall situation, to provide clearer responsibilities and contact information, or to increase safety through its use.

*“We also have an ECHR version in [name of city]. That’s not so complex. It’s more for acute patients and so on. I think, because they are very special patients, with whom there are also more than... Um, if I compare it with [name of the city], it is also sometimes used for very sick children, but there is no SOPPC team attached to it or something. So, I think this version for SOPPC teams is very good in terms of complexity and also well-structured, so you actually find what you need. (16_physician_MO)”*

Looking at the content of the ECHR system, a comment field was requested in which PPC professionals could discuss, make suggestions, or clarify questions relating to each view. The traceability of which person made which entry was expressed as particularly important. An always-visible “header” (short summary about the patient including name, date of birth, main diagnoses, allergies, advanced care plan) was considered helpful to provide a quick overview of the patients. It became clear that some of the content previously stated as necessary by PPC professionals were very setting specific. This meant that some views were not intuitively understood by everyone, since only people from particular settings were currently working with a similar view or tested it in the context of previously conducted CTA with the same project. If views had short informational sentences, these helped the participants to understand the view better.

Concerning an implementation of the ECHR system, participants noted that an introduction or training would be necessary to use the ECHR system and expected challenges in the transition from an analog to a digital system. It was also suspected that younger PPC professionals would be able to cope with the change to an ECHR system more quickly than older professionals.

Participants mentioned that some oral handovers cannot be replaced but could be supported by the ECHR system. Moreover, it is necessary to critically examine for which patients the ECHR system would offer an improvement. For patients with an oncological disease who are only cared for over a short period of time, for instance, the use of the ECHR system would make less sense than for children with complex chronic diseases and long periods of care.

The participants expected that not all PPC professionals would use the ECHR system with the same intensity. Some individuals might selectively use components of the ECHR system, while its success also depends on how intensively all stakeholders use it.

*“[…] I don’t think you can basically say the ECHR system is going to work well or not work well. But probably the ECHR of patient A works well because it is well-maintained, and you know you find the things. Patient B’s ECHR doesn’t work because it’s not maintained. (19_physician_MO)”*

### 3.2. MC 2—Start Page

Concerning the start page, in terms of functionality, participants brought up the need to be able to filter or sort the list of patients in some way.

*“[…] I only have four patients now, so I imagine I could have 350 patients or even 1500 because I’m a pediatrician, running a medical office. Then, I have the feeling that it could become relatively confusing. For me, it would be good if there was a search field, which I don’t see now. And secondly, maybe a sorting option, so that at least the ones where there is something new somehow always end up on top. Also, a kind of flag, similar to an email inbox, that it’s just printed in thick print when there’s something new in there (14_physician_PPCU+SOPPC)”*

In view of the content, the participants indicated that the user-related calendar was not necessary. The existing note option was positively evaluated by the participants.

### 3.3. MC 3—Contact History

In general, the contact history view was evaluated positively by some participants, as it allows all PPC professionals to see what things were discussed with the patient and their family. Referring to the form used to generate the entries in the contact history, there were some requests for improvement. Considering functionality, some participants wished to be able to see more than just three entries at a time, while others felt that three entries were sufficient.

Relating to the content view, participants stated that they found the structure of the online form too confusing. For example, they wished that frequently used fields (such as the comment field) were displayed higher up on the screen.

Initially, some participants were irritated by the logic of the contact history page, which only displays the name, time, and record system for the respective documented contacts to save loading time. The contents can only be viewed by unfolding. Some participants thought that no information was available or were unsure how to view it.

*“Uh-huh, okay. I just assumed that there was nothing documented there. Because for me, this “open to see content” was so unobtrusive that it was not visualizable at all for me at that moment. (14_physician_PPCU+SOPPC)”*

After an explanation, this logic was assessed as comprehensible and sensible. Nevertheless, the participants wished that the core data included further information, such as the profession and field of work of the person entering the data, as well as the location of the contact and the reason they were listed.

### 3.4. MC 4—Diagnoses and Findings

The diagnoses and findings view, which consisted of diagnoses, document collection, information on allergies, intolerances, and serious adverse events, as well as a timeline and vaccination status, was evaluated in a positive sense overall.

With respect to the diagnoses view, the participants wished for a better visualization of the diagnosis sorting, which would make the causality more comprehensible. Although the option of sorting the diagnoses by drag and drop was rated as positive, there was a desire to make clearer which diagnoses followed previous ones or were particularly relevant, by using bold print and indentation.

The participants found it positive to have access to many documents in the documents view (e.g., letters, laboratory results) and to be able to filter by category. However, they also wished that file naming was subject to a fixed rule when uploading to further increase clarity.

The past medical history view was perceived as helpful in providing an overview of the course of a child’s illness and treatment to date. Nevertheless, the participants would have liked to see more options, such as an unlimited view of all content instead of the current three-month view, the additional display of letters—stored with the respective application for an inpatient stay—and the option to see where outpatient or inpatient care took place (e.g., children’s hospice XY or clinic Z).

*“That’s when we get to know children for the first time, it often requires detective and detailed work to put all the information together chronologically first and then to understand the medical history. Often, the parents have thick folders where if you’re lucky, they have information. […] It’s very helpful, but it needs to be maintained so that things end up there. Because that’s the only way to access it. Somebody has to go through the trouble of saving the doctor’s letter or something, but if it works, it could be worth a lot. (10_physician_SOPPC)”*

It was mentioned that displaying all appointments in the medical history might limit clarity.

One participant suggested displaying a checkbox when entering appointments to specify that an appointment only appears in the medical history if it is relevant to it.

### 3.5. MC 5—Medication

Overall, the participants complimented the medication view and underlined the need for a common place for medication documentation:

*“No, it really doesn’t work well the way it currently is between the different settings. In our case, it’s the classic method, so to speak, clinic, children’s hospice, and SOPPC team. The pediatricians are somehow still quite far out. Yes, they keep completely different or other lists, which we usually don’t even know about. No, and even these three settings, inpatient children’s hospice, clinic, and SOPPC team. There are already always many, yes, problems, so to speak, that one medication plan or the other is not up to date. (…) Every doctor has the possibility, so to speak, to stop any medication. And of course, they have different ideas because different people have different ideas in their heads. And giving different dosages and so on and so forth. (12_nurse_SOPPC)”*

However, they also stated that counter-confirmation of medication orders by ECHR system users through PPC professionals from the PPCU or SOPPC teams could lead to difficulties and was time consuming. From the participants’ perspectives, the goal was that all persons could add content, although this could also be associated with difficulties (e.g., the fact that people enter medication late or medication can be discontinued by all users). A few participants found the option of control and a hierarchy for such decisions helpful and useful.

*“So, the diagnoses, of course, that’s also important, who has the sovereignty. But you’ve already clarified the rules. So that’s what I almost meant by the diagnoses. You can’t just make a diagnosis. Um, that must be justified. The general practitioner thinks the cold is important, and the palliative physician says: Nonsense, he has a cold every day. So, I think there must be a hierarchy. That’s what you do with the medications, there must also be. And a medication is requested. And this access to such a platform where everyone gets the same information about medications is worth its weight in gold. (18_physician_MO)”*

Toward the notice when a medication plan was uploaded, participants raised critical points relating to the timelines:

*“For example, let’s take Maxi who has been receiving amoxicillin from the SOPPC team since yesterday or the day before yesterday because of pneumonia. Now, the family doctor gets a letter from the cardiologist. The cardiologist says we have now increased the bisoprolol to 5 instead of 2.5 milligrams. Now, the primary care physician uploads the medication schedule three days after amoxicillin is scheduled. He uploads the schedule with the bisoprolol to 5 mg. But in the old medication plan, amoxicillin is not listed. Now the SOPPC team, or whoever, or the parents visiting the patient, want to have a look at the medication plan in order to give the medication, and either take the schedule from the family doctor with the correct bisoprolol medication but without amoxicillin, or the plan from the ECHR system and give the amoxicillin but not bisoprolol. The only case where this would be done correctly would be if the person looks at the plan in the ECHR system and the plan from the primary care physician in parallel. (19_physician_MO)”*

Participants wished for the option to create printouts from the medication view or to be able to save and make them available in analog form for their own documentation in other software, for the parents or for the nursing service providers. In their opinion, however, it should be more clearly visible for what reason a medication was started or discontinued, and the course of the medication should also be visible.

### 3.6. MC 6—Providers and Prescriptions

The providers and prescriptions view was also rated positively, as an overview of all involved PPC professionals is often not present. In terms of the content, it was stated that more specific information on assistive devices (e.g., which wheelchair, used for how long, responsible medical supply stores) is missing.

*“It would be quite useful for us if I could see what size bed he has and what mattress he uses if he uses a special one. Whether he has a wheelchair with an adapted seat shell. Whether he can see without the visual aids listed here. I don’t know if that shows up anywhere else. These are things like that from the care history. And whether he is dependent on the palatal plate. (03_nurse_PPCU)”*

Moreover, the participants identified that the area of performed therapies is currently missing and should be in this section. Additionally, the participants wished for the possibility to fill and save prescriptions digitally.

### 3.7. MC 7—Calendar

The participants found the use of the calendar intuitive and rated it as very helpful to avoid appointment overlaps or to obtain an overview of the current situation. They wished that the calendar had even more functions, such as the ability to invite people to appointments, to export appointments to their own calendar (Outlook), or to be able to assign different colors to appointments on different topics.

*“All dates are now highlighted in blue here. Maybe it would be good if I had different colors. For example, recurring appointments in one color or all physiotherapy appointments in another color with a legend that I can create myself. (11_nurse_PPCU)”*

### 3.8. MC 8—Treatment Process

Many participants rated the treatment process view as generally positive and helpful for the overview of previous successful or frustrating diagnostic or therapeutic attempts, as well as for a coherent reflection of their current work. Nevertheless, feedback was received that a field for documenting the treatment results should be renamed “current situation/results” for better tracking. Especially in the care of young palliative patients, there are often several hypotheses for one symptom, and several treatments are pursued in parallel. From the participants’ perspectives, this cannot be represented in a linear model. Rather, a structure like that of organizational charts with parallel strands is needed.

*“But the relation is not a one-to-one relation, it’s one-to-many. So, a symptom can have three different causes, which are approached in nine different ways. And sometimes you have to say what is the right hypothesis? You say, the child shows pain behavior. I don’t know exactly. Is it because the hip joint is not quite in the socket? Or is it because the child has a toothache? And then I say, working hypothesis one, toothache, working hypothesis two, hip dysplasia. Get the dentist, he says there’s nothing there at all. It’s perfectly fine. And then that one gets cancelled again. And then the others remain. But that can’t be mapped very well. (01_physician_SOPPC&PPCU)”*

The participants expected to be able to expand the fields where entries were made by clicking directly on them and not on an arrow in the margin.

### 3.9. MC 9—Messaging Function

The messaging function was rated positively to communicate with other PPC professionals. They emphasized that the ability to create groups was helpful, for example, to send a message to everyone who is caring for a child. There was confusion among some participants as to whether the messaging function was patient-related or user-related.

*“Participant: Am I still on Maxi’s ECHR here now? Or is this a general message? Do I still have to enter Maxi’s name in the subject field? Or does that happen automatically?*

*Interviewer: What would you think?*

*Participant: Well, the way I see it now, I think I would put “Maxi” in the subject line, that is [subject line]. Because it’s about Maxi. So, I would like to discuss something with her here, about Maxi. And I would write “Maxi” in the subject line.*

*Interviewer: Exactly. This message function is user-related, not patient-related. (04_nurse_PPCU)”*

### 3.10. MC 10—Methodology of the CTA

Overall, participants rated the CTA methodology as positive and indicated that they simultaneously found the CTA session to be a good introduction to the ECHR system and now felt well-prepared to use it. The implementation of the CTA approach via the remote session was also rated positively. However, participants from the PPCU and SOPPC teams found it challenging at times during the CTA session to differentiate between the ECHR system and the electronic medical records used daily.

*“I may sometimes think too strongly in our current system. And I think I’m not allowed to do that. Because the ECHR is meant for something else first. Just for exchange between the different settings and providers. (12_nurse_SOPPC)”*

## 4. Discussion

The aim of this study was to evaluate how potential users from the PPC setting perceived the prototype ECHR system and to identify their wishes related to an adaptation of the prototype. The CTA approach provided unique insights and refinements for the ECHR system while offering a multifaceted understanding of users’ perceptions of the ECHR system and how they would interact with the dataset under real-world conditions.

Participants generally rated the ECHR system as a useful tool to improve the information level of knowledge about patients, communication between PPC professionals, and the cooperative planning of patients’ treatments.

In this study, it became apparent that new challenges and questions arise regarding the logic of the ECHR system: (1) Who is responsible for the ECHR? (2) Who has what authority in terms of access to read and write? (3) How does the ECHR system interact with other systems?

One possible solution would be to designate one person per ECHR (e.g., the primary care physician) to ensure that the ECHR is properly maintained and always up to date. This person would assign the rights for each user in accordance with an agreement with the parents and the associated release from confidentiality. Depending on necessity, persons would be granted access to read and write some or all areas of the ECHR, either indefinitely or for a limited period. A counter-confirmation of the medication was evaluated diversely among the participants. In any case, it is crucial that it is always clearly visible that a new medication has been added to the medication plan and that no discrepancies arise because people first must confirm medications so that others can see them. Here, it is challenging to develop a future solution that takes into account the problem that remirroring of content leads to overwriting (see *2.2 Prototype ECHR*). A possible consideration is that entries can be made in all views by the ECHR system users and that these also appear as “real” entries, including the name and date of the person documenting. However, they will still appear as suggestions in the SOPPC and PPCU systems, so those PPC professionals will first confirm or deny whether a change and entry is still current when the child is in their care. In addition, the option requested by participants to add comments on individual pages, for example, to discuss a medication adjustment in a forum, could be helpful to reduce “competence wrangling” and the resulting reciprocal generation of entries. This clearly demonstrates the challenge of participatory design to realize the wishes of participants with technological capabilities while ensuring patient safety.

With respect to the content, as reported in other studies [7,33] participants highlighted the benefit of receiving timely, instantaneous information. In this regard, the possibility of a joint medication plan was particularly positively evaluated since the medication of PPC patients is very extensive [34]. Because of the frequent off-label use of medications in pediatrics in general [35], and in PPCs in particular [36], participants’ desires for information to justify the initiation or discontinuation of a medication is an important improvement to the ECHR system. It was found that 18% of all medical errors leading to an adverse drug event in inpatient care resulted from medication information not being available [30]. It is possible that such risks can be reduced by the ECHR system. In terms of content, the treatment process was found to be helpful as one of the major challenges of PPC is symptom control, which requires the interaction of all PPC professionals [3]. Due to the many years of support [6], it is hardly possible to trace the measures taken (successful or unsuccessful) without a systematic approach. However, it was rated as too linear and requires adaptation or new development. Smaller adjustments, such as changing icons and adding information texts, are required for other views. There were still views of specialized care and permission to access the ECHR system, which were not tested by the participants during the study. These views also require extensive testing by PPC professionals.

In view of the functions, the issue of assigning responsibility and integrated or federated access control has already been addressed in another study about shared health records [37]. Clarification of this issue is important to maintain clarity and correctness of the ECHR system. Participants in the study suggested that one of the PPC professionals should always be responsible for the ECHR system. This person should approve individuals’ access areas after consulting with parents and patients and ensure that information is always up to date. However, participants described it as challenging that all PPC professionals are heavily involved in their work and that this would mean additional effort. Due to the diversity of the systems used, the possibility of automatically transferring data from the systems of the medical offices will be critical. This difficulty is also already described in the literature [38]. However, the transfer of data from SOPPC teams and the PPCU was evaluated as helpful. Nevertheless, the participants wished for further functions (e.g., free text search) and content (e.g., information about therapies).

The participants and members of the research and development team rated the methodology of CTA as positive. Some studies have examined the effects of different think-aloud methods: one study reported on the comparison of two different think-aloud approaches [39]. In one of the approaches in the study, interviewers were instructed to be very reserved. In the second approach, interviewers could express appreciation (using “mm-hmm”), ask for clarifications, or offer support. The task performance of those who received feedback (second approach) was significantly better in the study: participants completed more tasks. The rating of the tested website in this study and the number of problems detected did not differ between the two groups [39]. Another study found that CTA with explicit briefing on the logic of the software resulted in a greater number of named problems in the areas of dialog, navigation, layout, and functionality. However, the problems named in this briefing had low severity compared to the problems named by users who had received only a neutral briefing prior to the CTA session that focused on the methodology of CTA [40]. In our study, we used a combination of the approaches found to be positive in both studies [39,40] and supported participants by showing appreciation and by offering assistance after some time had passed to solve problems on their own. We also developed a structured guide to explain the logic at various critical points in the software, as seen in the study by Zhao et al. (2012) [40]. The approach developed by combining these two approaches resulted in helpful feedback from the research and development teams’ perspectives.

Following this study, after the revision of the ECHR system based on the feedback received, further testing should take place. A frequently used approach for this is near-life testing, in which participants interact with actors as patients while testing the software [41,42]. The method and corresponding design of such a scenario would have to be adapted according to the use of the ECHR system. However, it offers the possibility to improve the ECHR system iteratively and ensure the benefit for PPC, as well as avoiding disadvantages for patients (e.g., due to incomplete information collection).

Another challenge will be the implementation of the ECHR. After the end of the project, the software producer will receive the rights for the beta version of the ECHR. After completion, there is the possibility of marketing. However, this implementation process should be carefully planned to be successful. One challenge is the funding of the software, which may have a benefit but is not legally obligatory to use. In addition, the support from hospitals, SOPPC teams, and healthcare administrations to use the ECHR is critical. As also named by the participants in the study, the time of all PPC professionals is limited and they are already very busy. For this reason, the participatory approach was critical to lowering barriers to use as much as possible. Now, external incentives need to be created to use the ECHR. Here, frameworks, such as the Consolidated Framework for Implementation Research (CFIR) [43], can be helpful and should be included in further research [44].

## 5. Limitations

A limitation of this study is the sample size. Despite intensive recruitment, it was not possible to recruit a larger sample. In particular, the recruitment of pediatricians and general practitioners from medical offices was very time consuming. By extending the recruitment and survey period by 3 weeks, 5 persons were recruited. The additional workload of the physicians in medical offices due to COVID-19 vaccinations probably had further effects here. A further extension of the recruitment and survey was not possible due to the project schedule. In terms of pure usability testing, the model of Virzi (1992) states that 80% of problems are detected by four or five participants and the most severe problems are noticed by the first participant [45]. We can state something similar for our study. Due to the special setting, it is therefore the content focus that could gain breadth through additional participants due to the diversity of patients and forms of care. Nevertheless, it was possible to achieve theoretical saturation and obtain a wide range of feedback

Unfortunately, some characteristics (Table 1) are missing due to nonreturn of the questionnaire. Surely it would have been interesting to get the characteristics from them. However, the crucial information for the present study is the fact that they had experience in the field of PPC, which was crucial as inclusion in the study. The other crucial information was the profession, which could be documented in all participants.

The people who agreed to participate are presumably those who are particularly interested in communicating with other PPC professionals and improving care. This may have had an impact on the assessment of the overall logic of the ECHR system. In addition, it can be assumed that these persons had a higher digital affinity and were less averse to digital forms of documentation than those who possibly refrained from participating for this reason.

In future studies, it is desirable that persons from other work areas (e.g., physiotherapists) could also provide feedback on the functioning and contents of the ECHR, as they make important contributions to holistic care. Moreover, the study only took place in Germany. This study can serve as a guide to provide a framework to developing similar systems in the health care systems of other countries.

## 6. Conclusions

The ECHR system met user requirements in different aspects but needs further improvement. This study demonstrated the need for iteration in the process of collaborative design processes. The development of complex software for PPC involves many different logics, contents, and functions that can be developed not only by interviewing potential future users and designing prototypes but also by using the software itself. A challenge of the study was the different participants with their diverse experiences, views, requirements, and habits. The functionality of the ECHR system was evaluated positively. Only minor adjustments are necessary here, such as free text search in several places or the possibility of opening and closing fields together. Regarding the content, some areas could be reduced and formulated more specifically to avoid a flood of information and reduce misunderstandings. The treatment process needs to be further adapted. In terms of the logic, from the point of view of the participants, adaptions are necessary so that entries can be made at all points via the ECHR system and content is not only automatically fed from other systems. The goals in view of the adaptation of the ECHR prototype are as follows: (1) the ECHR system should be clear; (2) it should support communication between PPC professionals; (3) it should have a benefit to the user beyond reproducing information; and (4) it should ensure that the information is correct and up to date.

The mere exchange of documents that currently takes place through ECHR systems [14,15] is certainly not sufficient in other fields with persons suffering from complex diseases. Therefore, the development of broader ECHRs should emerge based on the insights obtained here and the findings from the needs assessment.

However, meeting these challenges and working with users to develop solutions holds enormous potential to develop an ECHR system that makes the care of PPC patients safer and more dependable for all stakeholders.

## Figures and Tables

**Table 1:** Participants’ characteristics.

		PPCU (*n*) ^a^	SOPPC (*n*) ^b^	PPCU/SOPPC (*n*) ^c^	Medical Office (*n*) ^d^
Sex (*n*)	Female	5	3	3	1
	Male	1	1	1	4
Age in years (*M*)		40.5	47	45.5	59.4
Profession (*n*)	Nurse	5	4	-	-
	Physician	1	1	4	5
Years of work experience (*M*)		17.25	22 ^e^	21.3	30.0
Years of experience in current position (*M*)		9.8	11.5	11.6	18.7
Experience with electronic health records (*n*)	No	5	-	4	2
	Yes	1	4	-	1
Experience with electronic cross-sectional health records (*n*)	No	5	2	3	-
	Yes	1	2	1	3

^a^*n* = 6. ^b^ *n* = 5; (One nurse’s characteristics are missing due to the nonreturn of the questionnaire). ^c^ *n* = 4. ^d^ *n* = 5 (Two physicians’ characteristics are missing due to the nonreturn of the questionnaires). ^e^ This question was answered by three participants from the SOPPC teams (One nurse’s characteristics are missing).

## Data Availability

The corresponding datasets of this study are available from the corresponding author upon reasonable request.

## Supplementary Materials

The following are available online at https://www.mdpi.com/article/10.3390/children8100839/s1, File S1: Tasks of the CTA, File S2: Interview guide, File S3: Introduction to the CTA, File S4: Feedback on the adaptation of the software.

## Appendix A

Within the scope of the ELSA-PP project, an electronic patient record for the inpatient area of PPC, an electronic patient record for the outpatient area of PPC, and an ECHR system will be developed in a participatory approach with future users. Since the development of all components would exceed the time frame of the project, the developments are based on the already existing software “Information System Palliative Care” (ISPC) from the company smart-Q Softwaresystems GmbH—an outpatient record system for the palliative care of adults [46] (see https://www.smart-q.de/ed-portfolio/ispc/ (accessed on 21 September 2021–for further information)).
