# Supplementary material for: Participatory Design of an Electronic Cross-Facility Health Record (ECHR) System for Pediatric Palliative Care: A Think-Aloud Study
Source: Children (Basel). 2021 Sep 24;8(10):839. doi: 10.3390/children8100839 (PMC8534759; doi:10.3390/children8100839)
Supplement: Supplementary file 1 [file children-08-00839-s001.zip › S1 ¿C Tasks CTA.pdf]

Tasks ECHR

Guideline for testing the ECHR system in the ELSA-PP project

Presentation of the persona

- Maxi Sonne, ♂, *03/15/2001
- Residing with his parents in (name of city)
- Rough overview medical history:

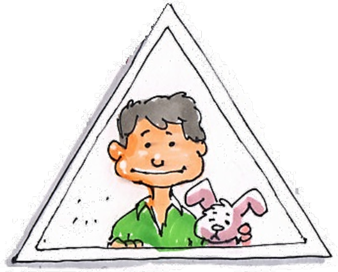

2003
Diagnosis Duchenne muscular dystrophy

2016
Straightening spondylosis due to neuromyopathic scoliosis

2017
Diagnosis dilated cardiomyopathy

2019
Increasing pain problems
→ support from outpatient care team

2020
Start of nocturnal home ventilation

2021
Inpatient stay for medication readjustment

Current reason for using the EFA

Maxi has been admitted to the pediatric palliative care unit for readjustment of medication due to increasing pain problems.

In the following, we simulate the possible exchange of information between the providers involved.

After the inpatient stay, Maxi is discharged back home and cared for there by the family doctor and the outpatient team. The ECHR system is used to exchange information between the pediatric palliative care unit, the outpatient pediatric palliative care team and the family doctor. Imagine you are caring for Maxi and using the ECHR system to exchange information.

Start page

You can now see the view of the ECHR system that you will get to after logging in. All patients for whose ECHR you are activated are displayed here. In addition, there are areas where personal notes can be entered and an overall calendar.

1. Get an overview of the start page.
2. Please select Maxi Sonne on the start page.
3. You will now be taken to the ECHR of Maxi Sonne. Please get an overview of the first view.

Contact history

In this view, columns are generated whenever an entry is made. These can be transferred from outpatient and inpatient electronic patient records or made in the ECHR system itself. The Admin tab contains information on where the entry came from (entered in the ECHR system or transferred from outpatient/inpatient care, by whom the entry was made, when and in which form). Content can be expanded on the left and will be visible.

1. Look at what entries were made on 01/01/2021.

2. Document with the plus button in the contact form that you have spoken with Maxi's parents today.

Diagnoses and findings I

1. Switch to the Diagnoses & Findings view.
2. View the overview of diagnoses.
3. Display all secondary diseases.
4. Get a brief overview in the other tabs.

Diagnoses and findings II

1. Switch to the overview of previous medical history and get an overview.
2. Display all entries from 01/01/2021 until today.
3. Search for the entry Contact form from 05/07/2021 and display the linked PDF.

Medication I

In this view, the main issue is that different medication plans exist and the ECHR system is intended to improve that the most up-to-date plan is available and that everyone works together on the medication.

1. Switch to the medication overview and get an overview.

2. Change the medication from Ibuprofen to 400mg 2x daily (any times).

Medication II

1. View the overview of on-demand medication.
2. View the crisis/emergency medication overview.
3. Upload a new PDF for the medication plan overview.
4. Check if the date of the medication plan has changed.

Provider and Prescription

1. Switch to the "Provider and prescription" view.
2. Get an overview of which assistive devices Maxi uses.
3. Maxi needs a new wheelchair. Find out which medical supply store is responsible.
4. The practice reports that she needs a new application for physiotherapy. The relevant document is in the ECHR. Please download this in order to send it.

Patient-related calendar

1. Switch to the calendar.
2. Check the calendar to see what appointments Maxi had after discharge from the children's and adolescent clinic on 30/04/2021.
3. Document that you have an appointment with the outpatient nursing service next week.

Treatment process

In this view, providers can consider together, symptom by symptom, what the hypotheses and options for treatment are. Here, they can then evaluate what results a treatment has produced.

1. Switch to the "Treatment process" view and get an overview.
2. View the information on the currently existing problem pain.
3. Please document "nausea" as a current situation. Formulate a hypothesis and a treatment.

Messaging function

1. Switch to the “messages” icon.
2. Send a message to any person and ask them to call you back.

Thank you very much for your participation!

Finally, we would like to conduct a short interview with you.

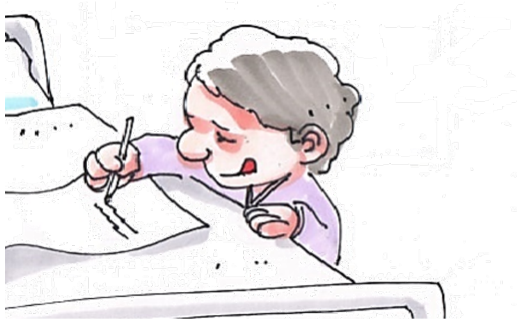

Illustrations from the Graphical Recording: Dagmar Gosejacob and Ralf Marczinczik
