# Supplementary material for: Participatory Design of an Electronic Cross-Facility Health Record (ECHR) System for Pediatric Palliative Care: A Think-Aloud Study
Source: Children (Basel). 2021 Sep 24;8(10):839. doi: 10.3390/children8100839 (PMC8534759; doi:10.3390/children8100839)
Supplement: Supplementary file 1 [file children-08-00839-s001.zip › S2 ¿C Interviewguide.pdf]

Appendix 2 - Interview guide

These are the questions that were asked to the users in the semistructured interview after the concurrent think aloud.

1. How would you describe your overall impression of the ECHR system?
2. How did you feel about the content?
    a. Is there anything you felt was missing?
    b. Is there anything you did not feel was necessary?
    c. What adjustments would you like to see beyond that?
3. How did you feel about the usability?
    a. Is there anything you liked about it?
    b. Is there anything you did not like?
4. Do you think that the ECHR system gives you a better overview of the patients?
5. Do you think that the ECHR system can improve the networking between the sectors (outpatient, inpatient)?
6. Do you think that the use of ECHR system can support the communication between the different sectors and pediatric palliative care professionals?
7. How do you think your colleagues (both from the same profession and from other professions and sectors) would evaluate the ECHR system and its content?
8. How did you feel about the testing methodology?
    a. Did you find it easy to verbalize your thoughts?
    b. Did you find the remote testing technique comfortable?
    c. If applicable, what made it difficult? What would have helped you?
