# Supplementary material for: Participatory Design of an Electronic Cross-Facility Health Record (ECHR) System for Pediatric Palliative Care: A Think-Aloud Study
Source: Children (Basel). 2021 Sep 24;8(10):839. doi: 10.3390/children8100839 (PMC8534759; doi:10.3390/children8100839)
Supplement: Supplementary file 1 [file children-08-00839-s001.zip › S3 ¿C CTA Introduction.pdf]

Appendix 3 – Brief introduction into the ECHR system

- Goals of the ECHR system:
  - ECHR system does not replace the own patient record for documentation, but is a supplement
  - Exchange across sectors
  - Availability of current information (emergencies, after sector change)
  - Common basis of information for informed treatment
  - More comprehensive information about patients
  - Possibility of communication between providers
- ECHR system serves as a common platform through which providers can exchange relevant patient information
- The ECHR system is based on information that has been documented in the inpatient and/or outpatient electronic patient record and are automatically transferred to the ECHR system (master data, medication etc.).
- In addition to the upload of documents, the exchange also includes the direct editing of individual contents.
- Access can be granted to all persons, it is possible to restrict the right of the users (read only, see only certain areas etc.)
- Various functions in the ECHR system, which will be considered while testing
- Focus on the possibility to post new findings, write messages, discuss things (for this purpose there is a comment function on some pages)
