# Supplementary material for: Participatory Design of an Electronic Cross-Facility Health Record (ECHR) System for Pediatric Palliative Care: A Think-Aloud Study
Source: Children (Basel). 2021 Sep 24;8(10):839. doi: 10.3390/children8100839 (PMC8534759; doi:10.3390/children8100839)
Supplement: Supplementary file 1 [file children-08-00839-s001.zip › S4-Feedback on the adaptation of the software.pdf]

Software view	Functions	Content	Logic
General aspects	**Suggestions:**<br>• free text search<br>• expand and collapse fields simultaneously<br>• transferring information from ECHR to medical office system<br>• Reminders for appointments/need for document update<br>**Critic:**<br>• Long loading times	**Suggestions:**<br>• comment field to discuss, make suggestions and clarify questions between PPC professionals<br>• traceability who made entries<br>• header with quick information about patients<br>**Critic:**<br>• Information overload due to transferred content from SOPPC and PPCU	**Suggestions:**<br>• possibility to add content in each view<br>• Permission system in which individual areas can be specifically released for users<br>**Critic:**<br>• Problem of jurisdiction
Start page	**Suggestions:**<br>• Filter and sort patient list	**Suggestions:**<br>• Remove user-related calendar	
Contact history	**Suggestions:**<br>• Opportunity to see more than three contacts	**Suggestions:**<br>• Adapt structure of the online form<br>• core data (profession, field of work) about the person entering data, the location of the contact and the reason for contact	
Diagnoses and findings	**Suggestions:**<br>• Better visualization of diagnosis sorting → causality<br>• File naming by fixed rule<br>• Unlimited view of all content despite three-month view<br>• Adding checkbox to appointments to make them appear in medical history	**Suggestions:**<br>• Display of letters in medical history<br>• Information about where outpatient or inpatient care took place<br>**Critic:**<br>• Displaying all appointments in medical history might limit clarity	
Medication	**Suggestions:**<br>• Everyone should be able to add content<br>• Possibility to create printouts / save medication view as document<br>**Critic:**<br>• Counterconfirmation could lead to difficulties	**Suggestion:**<br>• Reason why medication started<br>• Course of the medication	**Critic:**<br>• Opportunity to upload medication plan might lead to mistakes
Providers and pre-scriptions	**Suggestions:**<br>• Fill in and save prescriptions digitally	**Suggestions:**<br>• Additional information on assistive devices<br>• Information on performed therapies	

**Calendar**	**Suggestions:**<br>• inviting people to appointments<br>• Exportation of appointments to own calendar<br>• Assign different colors to appointments		
**Treatment process**	**Suggestions:**<br>• Expand fields by clicking on them	**Suggestions:**<br>• Field for treatment results should be named "current situation/results"	**Suggestions:**<br>• Structure similar to organizational charts with parallel strands
**Messaging function**			**Suggestions:**<br>• Make clear, whether messaging function is patient-related or user-related
